# Type 2 innate lymphoid cells from Id1 transgenic mice alleviate skin manifestations of graft-versus-host disease

**DOI:** 10.1186/s12865-021-00432-w

**Published:** 2021-07-13

**Authors:** Anand Srinivasan, Sandra Bajana, Aneta Pankow, Carrie Yuen, Rikin K. Shah, Xiao-Hong Sun

**Affiliations:** 1 Pediatric Hematology/Oncology and BMT, University of Oklahoma Health Sciences Center, Oklahoma City, OK USA; 2 Present addres: Pediatric Hematology/Oncology and BMT, The Hospital for Sick Children, University of Toronto, Toronto, ON Canada; 3 Arthritis & Clinical Immunology Research Program, Oklahoma Medical Research Foundation, 825 N.E. 13th Street, Oklahoma City, OK 73104 USA; 4 Hematology/Oncology and BMT, Department of Medicine, University of Oklahoma Health Sciences Center, Oklahoma City, OK USA

**Keywords:** Type 2 innate lymphoid cells, Graft-versus-host disease, Id1 transgenic mice, E proteins

## Abstract

**Background:**

Acute graft-versus-host disease (aGVHD) is one of the most common causes of morbidity for patients undergoing allogeneic stem cell transplantation. There is preliminary evidence that activated Group 2 innate lymphoid cells (ILC2s) from wild type (WT) mice reduces the lethality of aGVHD and is effective in treating lower gastrointestinal (GI) tract manifestations of aGVHD. This raises the prospect that ILC2s may be used for cell-based therapy of aGVHD but vigorous investigation is necessary to assess their impacts on different aspects of aGVHD. Genetically engineered mice which either express Id1 protein (Id1^tg/tg^), an inhibitor of E protein transcription factors or have E protein genes knocked out (dKO) in the thymus produce massive numbers of ILC2s, thus allowing extensive evaluation of ILC2s. We investigated whether these ILC2s have protective effects in aGVHD as WT ILC2s do using an established mouse model of aGVHD.

**Results:**

bone marrow transplant was performed by irradiating BALB/c strain of recipient mice and transplanting with bone marrow and T cells from the MHC-disparate C57BL/6 strain. We isolated ILC2s from Id1^tg/tg^ and dKO mice and co-transplanted them to study their effects. Our results confirm that activated ILC2s have a protective role in aGVHD, but the effects varied depending on the origin of ILC2s. Co-transplantation of ILC2s from Id1^tg/tg^ mice were beneficial in aGVHD and are especially helpful in ameliorating the skin manifestations of aGVHD. However, ILC2s from dKO mice were less effective at the protection and behaved differently depending on if the cells were isolated from dKO mice were pre-treated with IL-25 in vivo.

**Conclusion:**

These findings support the notion that thymus-derived ILC2s from Id1^tg/tg^ mice are protective against aGVHD, with a significant improvement of skin lesions and they behave differently from dKO mice in the setting of aGVHD.

**Supplementary Information:**

The online version contains supplementary material available at 10.1186/s12865-021-00432-w.

## Background

Allogeneic hematopoietic stem cell transplant (HSCT) is a potentially curative therapy for patients with many hematological and oncological diseases [1–4]. Graft-versus-host disease (GVHD) is the most common life-threatening complication of allogeneic HSCT [5]. Incidence of acute GVHD (aGVHD) ranges from 40 to 80% depending on the donor source [6, 7]. Patients with aGVHD are typically treated with glucocorticoids. Patients with aGVHD that do not respond to treatment with glucocorticoids have a poor long-term prognosis, with an overall survival rate of 5 to 30% [5]. Hence there is a need for new and innovative approaches to mitigate the symptoms of aGVHD.

Innate lymphoid cells (ILCs) have been identified as a distinct arm of the innate immune system and are considered innate counterparts of T lymphocytes [8]. It is divided into separate subtypes that encompassed not only natural killer (NK) cells, and lymphoid tissue inducer cells, but also non-cytotoxic ILC populations [9]. These non-cytotoxic family of cells include group 1 innate lymphoid cells (ILC1s), group 2 innate lymphoid cells (ILC2s) and group 3 innate lymphoid cells (ILC3s). These cells do not express signature molecules that typically define different hematopoietic lineages, thus considered lineage negative (Lin^−^). ILC1s, which resemble T helper 1 cells are cells that are capable of producing interferon-γ and tumor necrosis factor. NK cells are considered as a part of ILC1s, but mirror CD8^+^ T cells [10]. By contrast, ILC2s mimic T helper 2 cells and produce associated cytokines (including IL-4, IL-5, IL-9 and IL-13) [9]. These cells are known to promote type 2 allergic reactions but also facilitate tissue repair following influenza infection of the lung [11]. Lastly, RORγt^+^ ILC3s correspond to T helper 17 cells and are heterogeneous in both mice and humans [12]. There is growing evidence that ILC2s and ILC3s play a role in aGVHD and a protective role of ILC2s was shown in gut manifestations of GVHD in mouse models [13, 14].

ILCs are known to originate from common lymphoid progenitor cells and lymphoid primed progenitors in the bone marrow but recent reports also demonstrate the capacity of the thymus of ILC2 production [15–19]. The basic helix-loop-helix transcription factors encoded by E2A, HEB, and E2–2 genes are collectively called E proteins. E proteins are essential for the differentiation of both B and T cells and block the innate lymphoid fate in bone marrow and the thymus [18–20]. The functions of E proteins are inhibited by Inhibitor of differentiation (Id) proteins [21, 22]. We have previously described two distinct genetically engineered mouse strains that produce massive numbers of ILC2s in the thymus. The first strain was Id1^tg/tg^, where the Id1 transgene is expressed under the control of the lck proximal promoter. The transgene is known to be specifically expressed in the thymus at multipotent progenitor stage called ETP [23]. Id1^tg/tg^ mice have complete blockage of T cell differentiation but a 60-fold increase of ILC2s in thymus compared to wild-type mice (WT), as well as massive expansion of ILC2s in other tissues [18]. Another strain was created by specifically deleting two E protein genes (E2A and HEB) with the plck-Cre transgene (dKO), which begins to express at committed T cell precursor stages such as the CD4 and CD8 double negative 3 stage. These mice also produce enormous numbers of ILC2s in different organs [18, 19].

Given the different developmental origins of ILC2s in Id1^tg/tg^ and dKO mice, we sought to study if ILC2s produced in the thymus exhibit comparable properties as those previously reported for WT mice [14], and if Id1^tg/tg^ and dKO ILC2s behave differently in the context of aGVHD. Our study shows that ILC2s from Id1^tg/tg^ mice are protective from aGVHD, which suggest that these thymic ILC2s have commensurate properties as WT ILC2 thought to be made in the bone marrow or tissue resident ILC2s.

In contrast, ILC2s from untreated dKO mice displayed less beneficial effects on reducing the aGVHD manifestations of transplant recipients, whereas those from IL-25 pre-treated dKO mice exacerbated aGVHD. These results thus reveal distinct activities of ILC2s differentiated from T cell precursors relative to those derived from multipotent progenitors and the potential adverse impact of IL-25 exposure. Cumulatively, these findings illustrate the benefits of thymus-derived ILC2s in aGVHD while also emphasizing that ILC2s may behave differently based on how they are generated, which will be enlightening in future therapeutic applications.

## Materials and methods

### Mouse transplant models

Mouse models of aGVHD are well documented [24, 25]. Recipient BALB/c mice were irradiated with 780 cGy of irradiation using a Cesium 137 irradiator in 2 sessions 4 h apart. Donor CD45.1^+^ C57BL/6 mice were sacrificed on the day of transplant and bone marrow (BM) and spleen dissected out. Femur and tibia was used for marrow isolation. Spleen was sorted for T cells by selecting for cells that were negative for Peridinin-Chlorophyll-Protein/Cyanine5.5 (PerCP/Cy5.5) conjugated anti–mouse B220 (Biolegend, Cat. #103236) and anti–mouse CD25 (Biolegend, Cat. #102030) using MoFlo XDP. Recipients were administered 1 × 10^6^ of BM and 1 × 10^6^ sorted T cells through retro-orbital injections. Cohorts receiving ILC2s received 1 × 10^6^ of ILC2s. All animal experiments were performed according to protocols approved by the Institutional Animal Care and Use Committee policies at the Oklahoma Medical Research Foundation and in compliance with the ARRIVE guidelines.

### Isolation and activation of murine ILC2s

As described by Bruce et al., 8–12 week-old B6 mice were given 0.4 μg recombinant mouse IL-17E/IL-25 per day (Biolegend, #587306) by intra-peritoneal injection for 4 days [14]. On day 5, cells were isolated from the mesenteric lymph nodes and processed using Hank’s Balanced Salt Solution with 2% newborn bovine serum. ILC2s were isolated by sorting for Lin^−^Thy1.2^+^ cells. The lineage cocktail included anti-mouse FcεRI, anti-mouse B220, anti-mouse CD19, anti-mouse Mac1, anti-mouse Gr1, anti-mouse CD11c, anti-mouse NK1.1, anti-mouse Ter-119, anti-mouse CD3, anti-mouse CD8a, anti-mouse CD5, anti-mouse TCRβ, and anti-mouse TCRγδ antibodies and Thy1.2 was stained using antibodies conjugated with fluorescein isothiocyanate. Cells were cultured for 7 days in complete media with rIL-7 and rIL-33 (10 ng/ml) (R&D Systems and eBioscience, respectively), with the media replenished every 2 days.

### GVHD scoring and weight monitoring

All mice were monitored starting on day 1 with serial weight monitoring and GVHD scoring. GVHD scoring was based on previously described standard methods taking into account of weight loss, posture, activity, fur texture, and skin integrity [24, 25]. Each category accounts for a maximum score of 2, thus totaling to a maximum score of 10. Weight loss is a known feature of GVHD and most mice with severe GVHD manifest their symptoms as severe weight loss [26, 27]. Weight loss of over 10–25% is scored as 1 and that over 25% is scored as 2.

### Engraftment studies

For checking the rate of engraftment in our transplant models, we sacrificed select animals at the end of the experiments and tested the rate of engraftment in the BM. Since donor mice express the CD45.1 surface marker and recipients carry the CD45.2 marker, BM cells were stained using anti-mouse CD45.1 antibody conjugated with Phycoerythrin and anti-mouse CD45.2 antibody conjugated with Pacific Blue. Subsequently, they were analyzed by flow cytometry on an LSRII machine (BD Biosciences).

### Statistical analysis

All analyses were done using Prism software. For weights and GVHD scores, *p* values were obtained by using two-way ANOVA. All weights of mice were standardized as 100% on the day of transplant. Weight loss for each mouse was plotted as a percentage of their weight on day 0 to standardize variations of baseline weight of mice.

## Results

### Preparation of ILC2s for co-transplantation

**Fig. 1:**
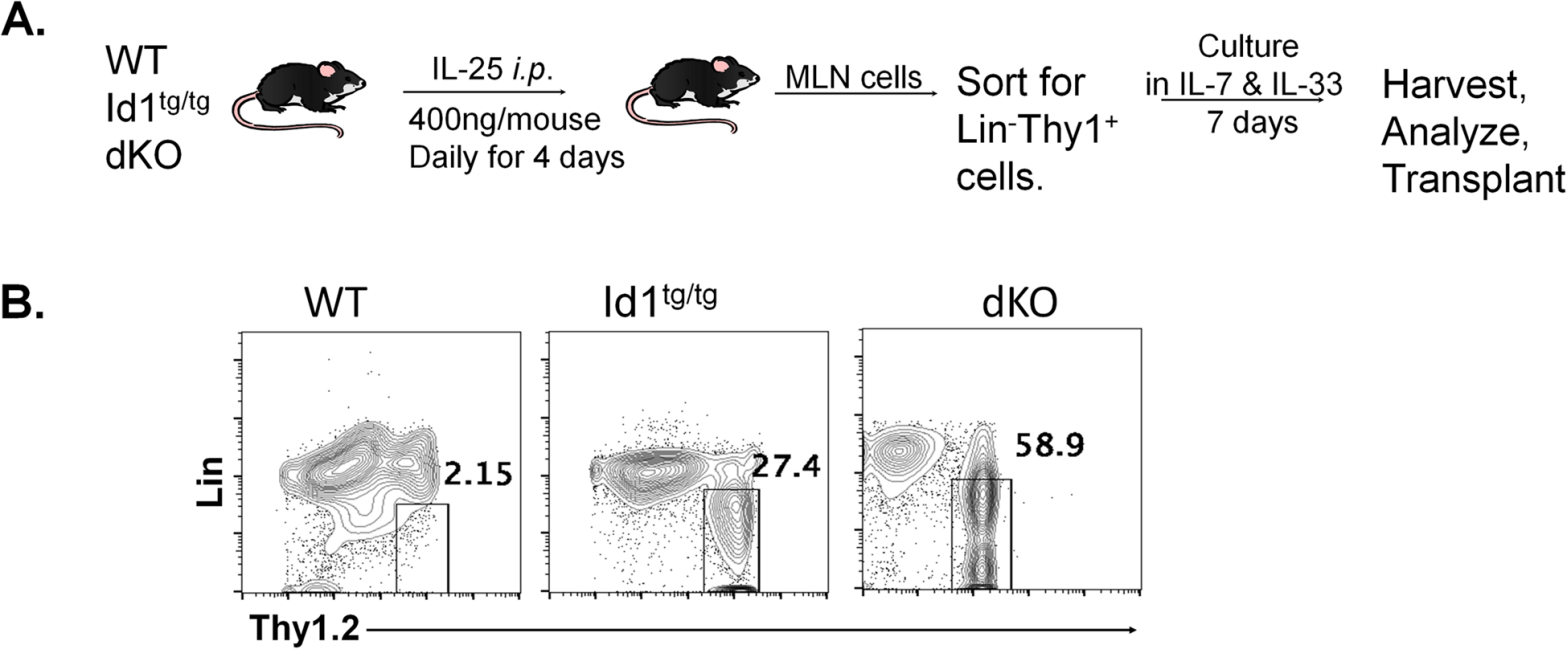
Preparation of ILC2s for transplantation. (A) Experimental scheme of the transplant experiments. (B) Gating strategy for sorting ILC2s using flow cytometry from the mesentery lymph nodes of IL-25 treated mice of indicated strains

Bruce et al. have previously reported the beneficial effects of WT ILC2s on gut manifestations of aGVHD by using cells isolated from C57BL6 mice. Both Id1^tg/tg^ and dKO mice, also on the C57BL6 background, generate a large amount of ILC2s in the thymus due to down-regulation of E protein transcription factors, which suppress the ILC2 fate. However, ILC2s from Id1^tg/tg^ and dKO mice arise from multipotent progenitors and committed T cell precursors, respectively [18, 19]. To prepare ILC2s for transplant, we followed the experimental schema reported by Bruce et al. (Fig. 1A) and sorted lineage-negative and Thy1^+^ cells from the mesenteric lymph nodes after the mice were treated with 400 ng of IL-25 per day for 4 days (Fig. 1B) [14]. These sorted cells were then propagated in 10 ng/ml IL-7 and IL-33 for 7 days. This resulted in approximately 10-fold expansion of ILC2s. It is of note that we were usually able to obtain fewer than 20,000 Lin^−^Thy1^+^ cells per treated WT mouse whereas about 10^5^ and 10^6^ such cells from Id1^tg/tg^ and dKO mice, respectively. Therefore, we could easily set up cohorts of at least 5 recipients of 10^6^ Id1^tg/tg^ or dKO ILC2s in each transplant session. However, we only had enough WT ILC2s from 10 treated donor mice for 1–2 recipients (10^6^ cells per recipient), thus making the transplant cohort difficult to establish.

In addition to ILC2s, we sorted B220^−^CD25^−^ splenocytes from C57BL6 mice to enrich for T cells while removing B cells and T regulatory cells. Whole bone marrow cells (BM) were used for each recipient to rescue hematopoiesis. We reasoned that T cell depletion from the bone marrow was unnecessary because most of the transplant cohorts received 10^6^ donor T cells and the BM alone group did not have long-term engraftment to cause GVHD (supplemental Fig. 1). Unfortunately, due to the lack of a donor-specific marker, we were not able to localize donor ILC2s in the recipients.

### ILC2s from Id1^tg/tg^ and dKO mice can be activated by IL-25 and IL-33

**Fig. 2:**
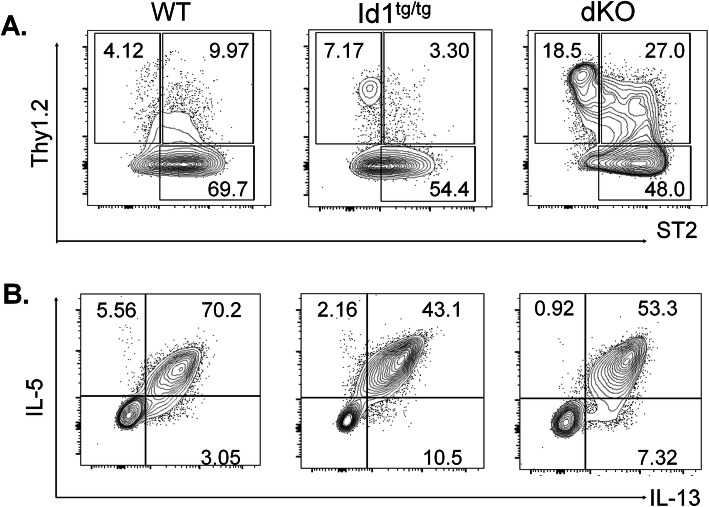
Expanded ILC2s from Id1^tg/tg^ and dKO mice display ILC2 characteristics. (A) Expression of ILC2s surface markers, Thy1 and ST2, on sorted cells after being cultured in IL-7 and IL-33 for 7 days. (B) IL-5 and IL-13 production by cultured cells detected using intracellular staining after stimulation with PMA and ionomycin plus a Golgi block, monensin, for 3 h

To assess the purity of our ILC2 preparations, we stained the cells for the expression of Thy1 and ST2 markers at the end of the 7-day culture (Fig. 2A). Although Thy1^+^ cells were placed in culture, incubation for 7 days led to the down-regulation of Thy1 expression, which is not unusual. Consistent with their response to IL-33, the majority of the cells expressed ST2, a component of the IL-33 receptor.

To determine the functionality of the ILC2 preparations, we tested their ability to produce ILC2 signature cytokines upon stimulation with PMA and ionomycin in the presence of a Golgi blocker, monensin, for 3 h. Intracellular staining for the expression IL-5 and IL-13 were then carried out. Cells from both Id1^tg/tg^ and dKO mice produced these cytokines as avidly as WT ILC2 controls (Fig. 2B), suggesting that these cells possess a key feature of ILC2s. This is consistent with our previous findings using lung ILC2s from WT, Id1^tg/tg^ and dKO mice [19]. Although PMA is a strong stimulator that might elicit non-physiological responses, our previous experience showed that stimulation with PMA and ionomycin only increased the magnitude of IL5/IL-13 expression [19].

### ILC2s from Id1^tg/tg^ mice are beneficial in GVHD

The aGVHD model was established by transplanting 10^6^ BM cells with or without equal numbers of C57BL/6 T cells plus or minus ILC2s into lethally irradiated BalB/c mice. Because C57BL/6 and BalB/c mice carry MHC haplotypes of b and d, respectively, GVHD was readily detectable in the transplant recipients. However, over 75% of the recipients survived up to 45–60 days and no difference in survival rates were observed among different groups of recipients, which comprise the recipients receiving BM only (BM), BM and mismatched T cells (BM + T) or BM and mismatched T cells plus Id1 ILC2s (BM + T + ILC2). WT ILC2s were also used in parallel co-transplantation experiments as controls.

**Fig. 3:**
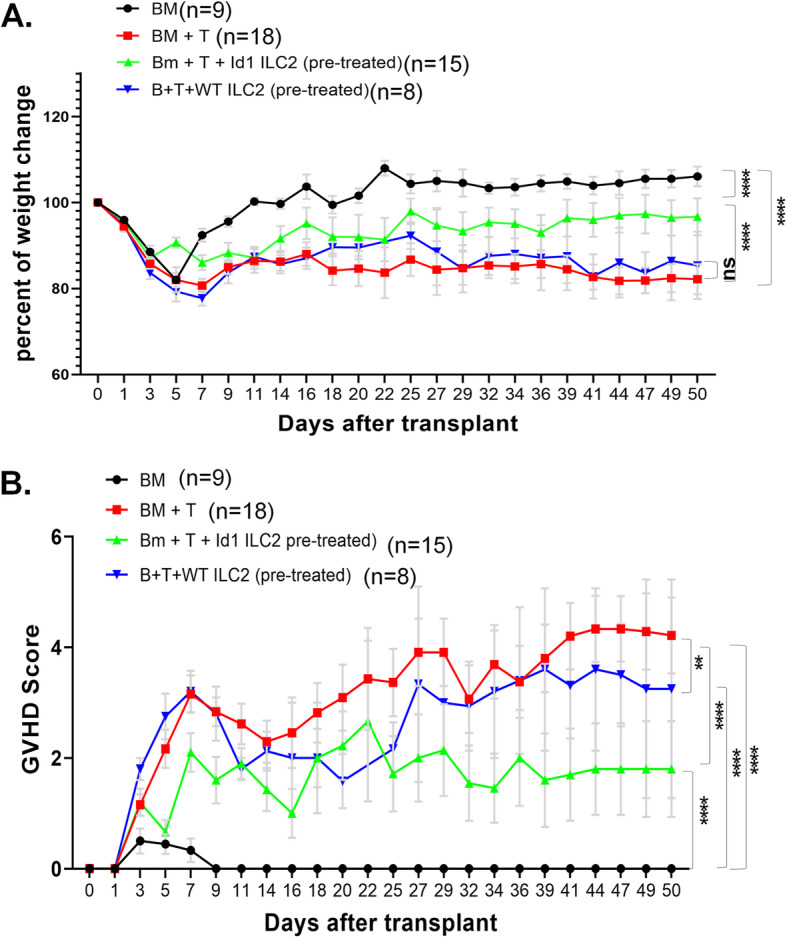
ILC2s from Id1^tg/tg^ mice have beneficial effects on aGVDH. Weight loss and GVHD scores of indicated cohorts of transplant recipients. Black circles represent mice that received BM alone (*n* = 9), red squares represent mice that received BM and sorted T cells (*n* = 18), and green and blue triangles represent the group that received BM, T cells and ILC2s from IL-25 treated Id1(*n* = 15) and WT (*n* = 8) mice, respectively. (A) Weight change is shown as a percent of baseline weight. (B) Cumulative GVHD scores are calculated based on weight loss, posture, activity, fur texture and skin integrity with the maximum score being 10. Values represent means and error bars represent the SEM. Two way ANOVA was used for assessing statistical significance. ** *p* < 0.01, **** *p* < 0.0001. Data is cumulative of 3 experiments

Weight changes were quantified as the percent of the initial weight. While the BM only group regained weight after overcoming the effect of irradiation, the BM + T group continued to exhibit weight loss with a mean weight of 85.6% of the original (*p* < 0.0001) (Fig. 3A). Co-transplantation with Id1 ILC2s alleviated the weight loss to 94.3% (*p* < 0.0001) of the initial weight. In contrast, WT ILC2s slightly improved weight loss (by 1.2%) compared to BM + T but the difference was statistically insignificance due to a small cohort of mice receiving WT ILC2s.

The GVHD score consists of 5 criteria: activity, posture, fur texture, and skin integrity as described by van den Brink et al. [26]. Within each category, a score of 1 or 2 is assigned based on the degree of alteration. For example, a weight loss by 10–25% is deemed a score of 1 and that of over 25% is quantified as 2. The maximum GVHD score is therefore 10, which represents the worst disease. When analyzing the GVHD scores of the three groups mentioned above, a similar pattern emerged where the mean score of the BM only group was 0.08 compared to the BM + T group which was 3.04 (*p* < 0.0001) and the BM + T + Id1 ILC2 group was reduced to 1.58 (*p* < 0.0001) (Fig. 3B). Co-transplantation of WT ILC2s also significantly decreased the mean score to 2.58 (*p* = 0.0013). Taken together, our data support the notion that Id1 transgenic ILC2s have protective effects on aGVHD.

### ILC2s from dKO mice have complex effects on GVHD

**Fig. 4:**
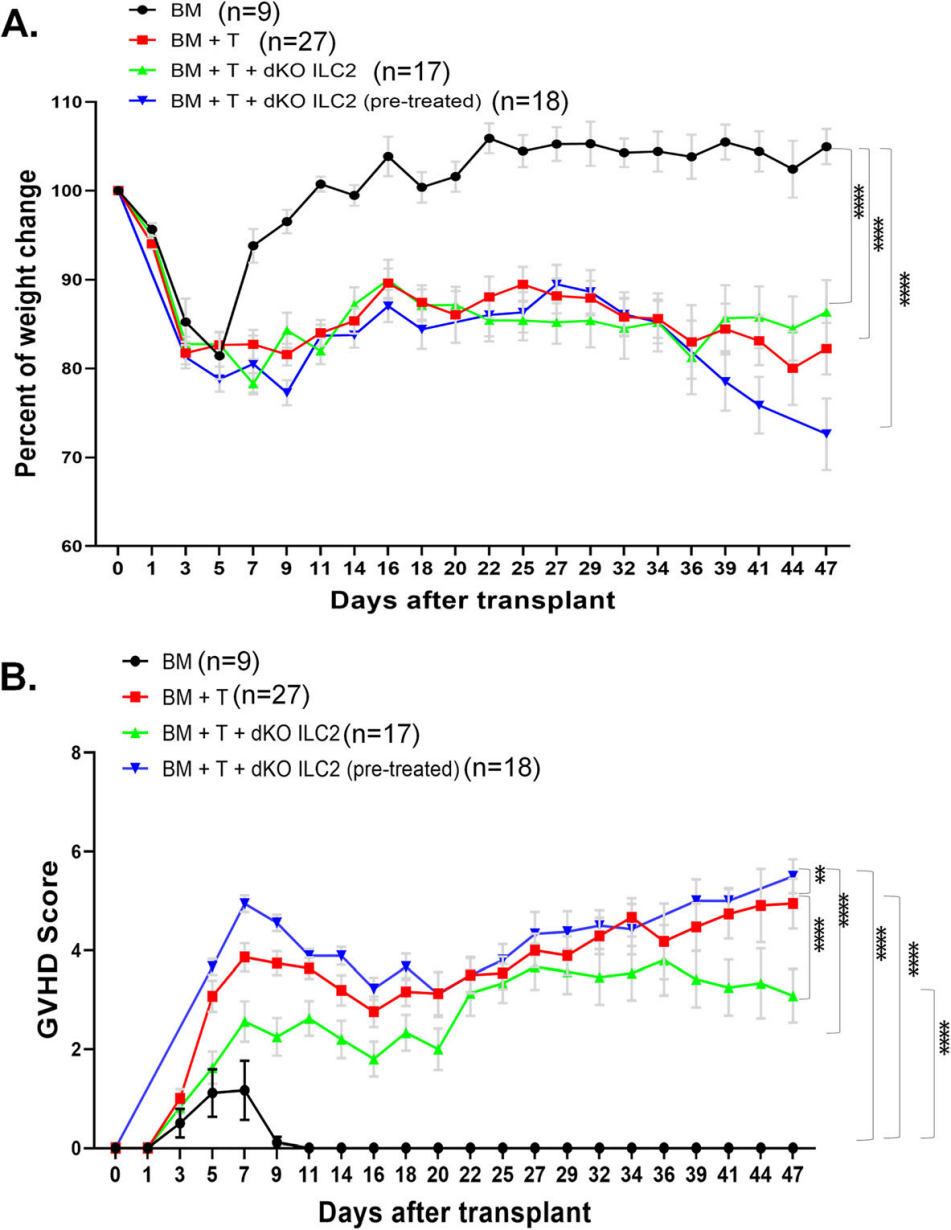
ILC2s from dKO mice have complex effects on aGVDH. Weight loss and GVHD scores of indicated cohorts of transplant recipients. Black circles represent mice that received BM alone (*n* = 9), red squares represent mice that received BM and sorted T cells (*n* = 27), and green and blue upward and downward triangles represent the groups that received BM, T cells and ILC2s from dKO mice treated without (*n* = 17) and with IL-25 (*n* = 18), respectively. The graphs are as described for Fig. 3. (**A**) Weight change is shown as a percent of baseline weight. (**B**) Cumulative GVHD scores are calculated based on weight loss, posture, activity, fur texture and skin integrity with the maximum score being 10. Values represent means and error bars represent the SEM. Two way ANOVA was used for assessing statistical significance. ** *p* < 0.01, **** *p* < 0.0001. Data is cumulative of 3 experiments

dKO mice have their E protein genes ablated at the committed T cell precursor stages and a large number of ILC2s accumulated throughout the body including mesenteric lymph nodes (MLNs). We were therefore able to evaluate the effects of these ILC2s from MLNs with or without pre-treatment with IL-25, which was intended to expand ILC2s in vivo. As described for Id1^tg/tg^ ILC2s, the mesenteric lymph nodes were processed and ILC2s were cultured for 7 days. The majority of the transplant recipients survived and no significant differences were observed among the different recipient groups (data not shown). Comparing to mice receiving BM only, both the BM + T and BM + T + dKO ILC2 groups showed significant weight loss (around 84–86% of the initial weight, *p* < 0.0001) (Fig. 4A). Unlike Id1 ILC2s, dKO ILC2s isolated from mice with or without IL-25 pre-treatment did not significantly alleviate weight loss relative to the BM + T group.

When analyzing the overall GVHD scores, dKO ILC2s from mice without IL-25 pre-treatment improved the scores compared to the BM + T group (mean scores of 2.53 vs. 3.41, *p* < 0.0001) (Fig. 4B). However, dKO ILC2s from IL-25 treated mice slightly worsened the scores (means scores of 3.80 vs. 3.41, *p* = 0.004). Together, the results show that dKO ILC2s have limited beneficial effects in aGVHD compared to Id1 ILC2s and strikingly, IL-25 treatment of dKO mice generated ILC2s with adverse impacts in the setting of aGVHD.

### Alleviation of skin lesions by Id1 transgenic ILC2s

**Fig. 5:**
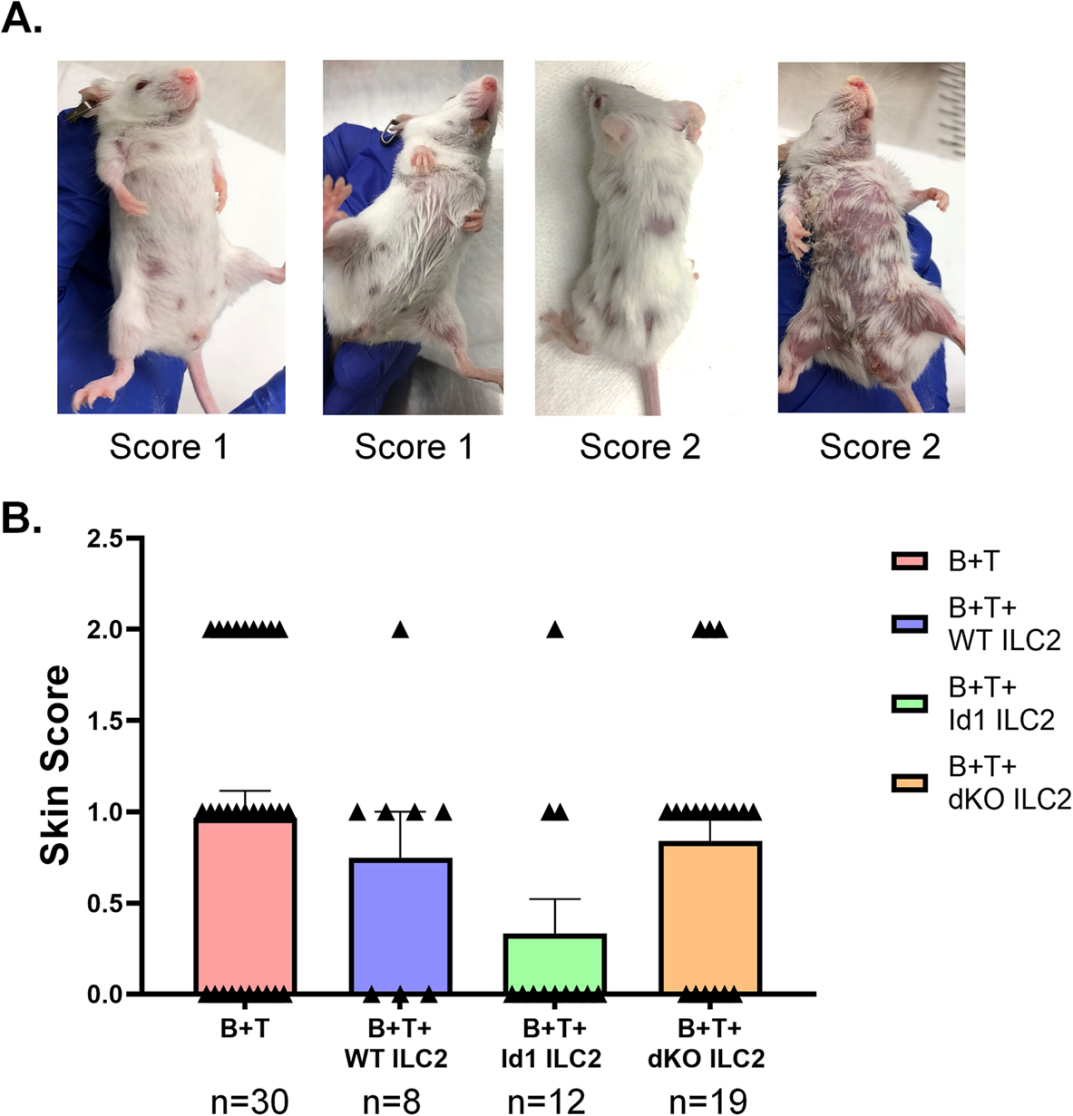
ILC2s ameliorate skin lesions. (**A**) Examples of skin lesions with a score of 1 or 2. (**B**) Average scores of skin integrity in indicated cohorts. The maximum skin integrity score observe in each animal was recorded. Data are cumulative from 3 experiments. Mean scores are presented as bars and each data point is shown by a symbol. Unpaired Student’s test was used to determine the statistical significance

One of major score-driving phenotypes was the development of severe skin lesions as evidenced by areas of denudation, dermatitis and multiple areas of tail hemorrhages in recipients of MHC-mismatched T cells (Fig. 5A). It should be noted that the conventional scoring scale for skin lesions. As shown in Fig. 5A, this is not quantitative enough to accurately reflect the varying degree of skin defects. Nevertheless, w compared the maximum scores of skin integrity in mice which survived the first three weeks after transplant in different cohorts. Consistent with the impact of ILC2s on the overall GVHD scores, ILC2s from Id1^tg/tg^ mice alleviated the skin lesion significantly compared to the B + T group (mean scores of 0.97 versus 0.33, *p* = 0.02) (Fig. 5B). WT ILC2s slightly reduced the mean skin scores by 0.22 but the difference was deemed statistically insignificant. In contrast, co-transplantation with ILC2s from IL-25 treated dKO mice did not significantly reduce the skin integrity score. ILC2s from dKO mice without IL-25 treatment also did not have any protective effects (data not shown).

## Discussion

Type 2 innate lymphoid cells are emerging as important players in allergic and autoimmune disorders [28–30]. With preliminary evidence of some protective effect of activated ILC2s in GVHD from mouse and human studies [13, 14], we sought to see how ILC2s from diverse sources would behave in the setting of GVHD. Here, we show that ILC2s from Id1^tg/tg^ mice when extensively activated, are also protective against the effects of GVHD. Both scores and weight loss from GVHD were ameliorated by the addition of ILC2s from Id1^tg/tg^ mice with a remarkable improvement in the skin manifestations of GVHD. This compares favorably to previous studies showing that WT ILC2s that are similarly activated exhibit protective effects on the gastrointestinal manifestations of GVHD [14]. In our study, WT ILC2s exhibit minor protective effects compared to published data. This may be due to differences in the approaches used in ILC2 and T cell preparations as discussed below. Nevertheless, the data suggest that ILC2s from Id1^tg/tg^ mice are as efficacious, if not more so, as WT ILC2s.

It should also be noted that the aGVHD detected in our study did not lead to high incidence of lethality of the recipient mice in less than 30 days as described by Bruce et al. [14]. This discrepancy may be due to the different approaches used in isolating the cells for transplantation. While Bruce et al. relied on magnetic depletion of unwanted splenocytes and mesentery lymph node cells to isolate T cells and ILC2s, respectively, we use cell sorting to ensure the purity of the desired cell populations. If the T cell preparations after negative selection contained antigen presenting cells, they may boost the activity of the mismatched T cells. Indeed, when we used total splenocytes containing equivalent numbers of T cells, we observed more severe disease and lethality but with a substantial variability. However, the prolonged survival of the recipients allowed us to assess the skin manifestation, which appear at later time points.

One caveat to be noted is that the protective effects were observed after extensive activation of ILC2s by IL-25 and IL-33 in vivo and in vivo. Another caveat is that these effects were observed at high doses of ILC2s which are supra-physiological levels. Co-transplantation with 10-time fewer ILC2s did not yield any protective effects (data not shown). However, this observation does suggest that Id1^tg/tg^ ILC2s behave similarly to WT ILC2s in the setting of GVHD, and may potentially be of therapeutic value [14] as a cellular therapy in the treatment of GVHD if properly expanded.

Remarkably, ILC2s from dKO mice behave distinctly from Id1^tg/tg^ or WT ILC2s, which differentiate from multipotent progenitors in the thymus or bone marrow respectively. dKO ILC2s originate from committed T cell precursors but they exhibit the basic characteristics of ILC2s by producing IL-5 and IL-13 in response to stimulation (Fig. 2) [19]. IL-13 was previously shown to be essential for the protective effects but the ability of dKO cells to produce IL-13 did not equip them with such a capacity [14]. Amphireglin has also been implicated in the protection from aGVHD since *Areg*^−/−^ ILC2s were somewhat less efficient in improving the survival of recipient mice co-transplanted with BM and mismatched T cells [14]. Our preliminary data showed that mature ILC2s from MLN of Id1^tg/tg^ and dKO mice produced similar levels of Amphiregulin after cultured in IL-2, IL-7, IL-25 and IL-33 for 5 days (data not shown). dKO ILC2s in small intestine also expressed comparable levels of *Areg* as their WT counterparts as determined in RNA sequencing studies. It is therefore unclear why dKO ILC2s do not exhibit protective effects as seen in Id1^tg/tg^ ILC2s. It is also worth noting that IL-25 treatment of dKO mice made the ILC2s isolated from their mesenteric lymph nodes exert adverse effects in the context of aGVHD. IL-25 is known to stimulate the generation of inflammatory ILC2s, and thus may render dKO ILC2s adopt inflammatory properties [31]. It is perplexing why these properties are not conferred on WT or Id1 ILC2s. It is tempting to hypothesize that dKO ILC2s may be more flexible in their cell fate and prone to induction to an inflammatory state, in stark contrast to WT or Id1 ILC2s.

With regard to the cellular mechanisms whereby WT ILC2s exert the protective function, Co-transplantation of WT ILC2s has been shown to be associated with reduction of the differentiation of donor Th1 and Th17 cells as well as the accumulation of myeloid suppressor cells [14]. The distinct function of Id1^tg/tg^ and dKO ILC2s will facilitate further investigation of the immunological manifestations in aGVHD.

In summary, we present aggregate evidence that Id1^tg/tg^ mice ILC2s behave similarly to WT mice in the setting of aGVHD, strengthening the notion that ILC2s are beneficial in GVHD [14]. However, if ILC2s are to be used as a potential cellular therapy for GVHD, our data also indicates that one should be cognizant of the varying properties of ILC2s generated in distinct manners and treated with a variety of stimuli. Careful consideration should be taken to avoid any adverse effects that may arise during the mass production of ILC2s in vitro, which is necessary for any regimen of cell therapy. Though ILC2s appear to be a promising candidate in the management of GVHD, further studies are essential before ILC2s can be considered as a candidate for manipulated cellular therapy in the clinical management of GVHD.

## Supplementary Information

**Additional file 1.**

## Data Availability

The datasets used and/or analyzed during the current study are available from the corresponding author on reasonable request.

## Abbreviations

aGVHD: Acute graft-versus-host disease,
BM: Bone marrow
dKO: E protein double knock-out mice
GVHD: Graft-versus-host disease
HSCT: Hematopoietic stem cell transplant
Id1^tg/tg^: Inhibitor of DNA binding 1 transgenic mice
Id: Inhibitor of differentiation
ILCs: Innate lymphoid cells
ILC1s: Group 1 innate lymphoid cells
ILC2s: Group 2 innate lymphoid cells
ILC3s: Group 3 innate lymphoid cells
NK: Natural killer
WT: Wild-type
