# Supplementary figures and images for: Type 2 innate lymphoid cells from Id1 transgenic mice alleviate skin manifestations of graft-versus-host disease
Source: BMC Immunol. 2021 Jul 13;22:46. doi: 10.1186/s12865-021-00432-w (PMC8278660; doi:10.1186/s12865-021-00432-w)

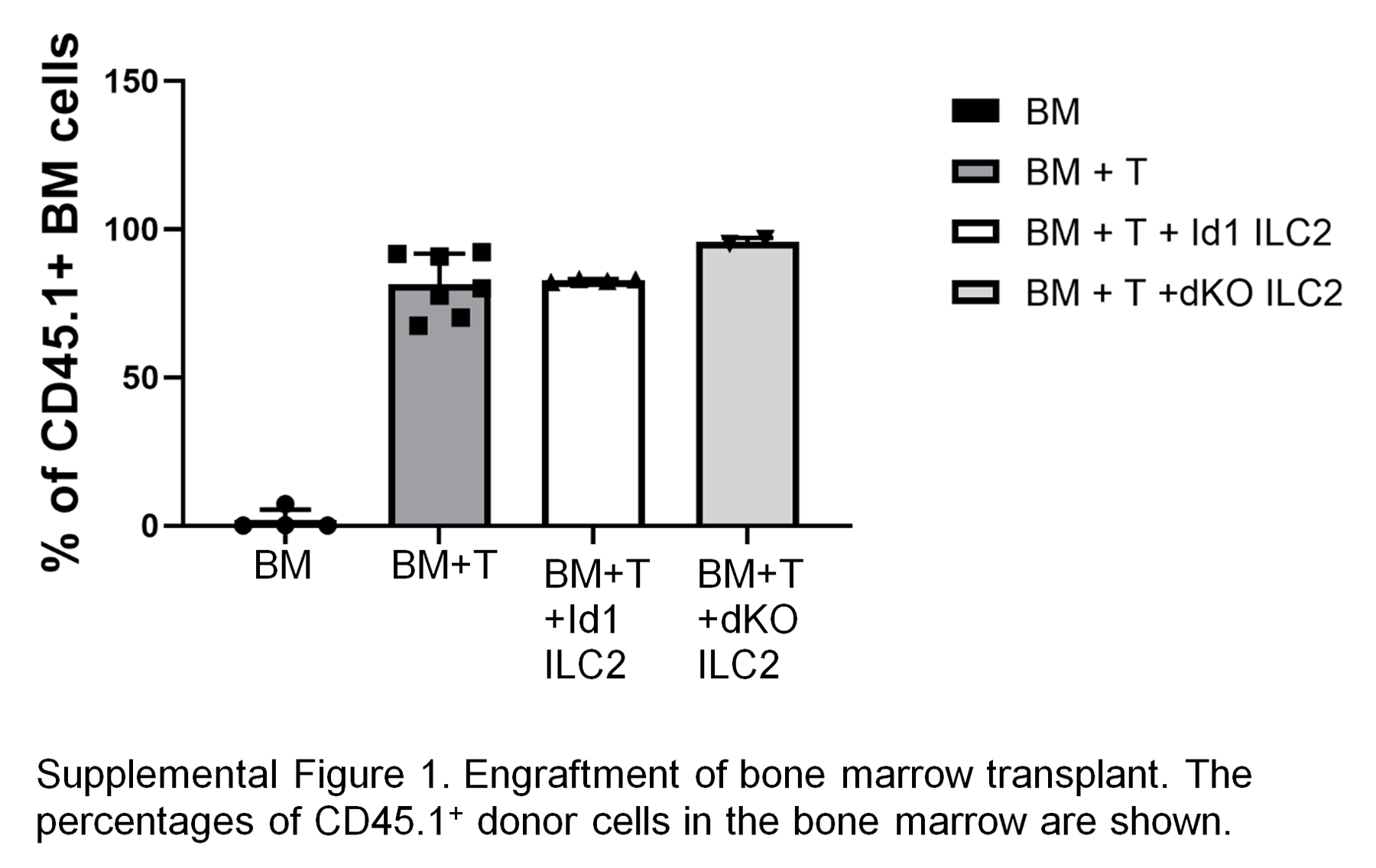

Supplement: Supplementary file 1 — Additional file 1. [file 12865_2021_432_MOESM1_ESM.tif]
